# Development and validation of predictive model based on deep learning method for classification of dyslipidemia in Chinese medicine

**DOI:** 10.1007/s13755-023-00215-0

**Published:** 2023-04-06

**Authors:** Jinlei Liu, Wenchao Dan, Xudong Liu, Xiaoxue Zhong, Cheng Chen, Qingyong He, Jie Wang

**Affiliations:** 1 Department of Cardiology, Guang’anmen Hospital, China Academy of Chinese Medical Sciences, Beijing, 10053 China; 2 Dermatological Department, Beijing Hospital of Traditional Chinese Medicine, Capital Medical University, Beijing, 100010 China; 3 Beijing University of Chinese Medicine, Beijing, 100029 China; 4 Xi’an Jiaotong University, Xi’an, 710049 China

**Keywords:** Dyslipidemia, Deep learning, Prediction model, Diagnostic factors, Traditional Chinese medicine

## Abstract

**Backgrounds:**

Dyslipidemia is a prominent risk factor for cardiovascular diseases and one of the primary independent modifiable factors of diabetes and stroke. Statins can significantly improve the prognosis of dyslipidemia, but its side effects cannot be ignored. Traditional Chinese Medicine (TCM) has been used in clinical practice for more than 2000 years in China and has certain traits in treating dyslipidemia with little side effect. Previous research has shown that Mutual Obstruction of Phlegm and Stasis (MOPS) is the most common dyslipidemia type classified in TCM. However, how to compose diagnostic factors in TCM into diagnostic rules relies heavily on the doctor's experience, falling short in standardization and objectiveness. This is a limit for TCM to play its advantages of treating dyslipidemia with MOPS.

**Methods:**

In this study, the syndrome diagnosis in TCM was transformed into the prediction and classification problem in artificial intelligence The deep learning method was employed to build the classification prediction models for dyslipidemia. The models were built and trained with a large amount of multi-centered clinical data on MOPS. The optimal model was screened out by evaluating the performance of prediction models through loss, accuracy, precision, recall, confusion matrix, PR and ROC curve (including AUC).

**Results:**

A total of 20 models were constructed through the deep learning method. All of them performed well in the prediction of dyslipidemia with MOPS. The model-11 is the optimal model. The evaluation indicators of model-11 are as follows: The true positive (TP), false positive (FP), true negative (TN) and false negative (FN) are 51, 15, 129, and 9, respectively. The loss is 0.3241, accuracy is 0.8672, precision is 0.7138, recall is 0.8286, and the AUC is 0.9268. After screening through 89 diagnostic factors of TCM, we identified 36 significant diagnosis factors for dyslipidemia with MOPS. The most outstanding diagnostic factors from the importance were dark purple tongue, slippery pulse and slimy fur, etc.

**Conclusions:**

This study successfully developed a well-performing classification prediction model for dyslipidemia with MOPS, transforming the syndrome diagnosis problem in TCM into a prediction and classification problem in artificial intelligence. Patients with dyslipidemia of MOPS can be accurately recognized through limited information from patients. We also screened out significant diagnostic factors for composing diagnostic rules of dyslipidemia with MOPS. The study is an avant-garde attempt at introducing the deep-learning method into the research of TCM, which provides a useful reference for the extension of deep learning method to other diseases and the construction of disease diagnosis model in TCM, contributing to the standardization and objectiveness of TCM diagnosis.

## Introduction

Dyslipidemia is a medical condition that refers to an abnormal level of lipid metabolism, including high levels of total cholesterol (TC), Triglyceride (TG), and low-density lipoprotein cholesterol (LDL-C) and low levels of high-density lipoprotein cholesterol (HDL-C) [1, 2]. Dyslipidemia is recognized as a prominent risk factor for cardiovascular diseases (CVD) [3–6] and one of the primary independent modifiable factors of diabetes [7] and stroke [8]. Clinical observations and epidemiological studies have shown that dyslipidemia increases the risk of cardiovascular disease events [9–11]. Therefore, the effective prevention and treatment of dyslipidemia are of great importance [12].

Statins can significantly improve the prognosis of dyslipidemia. However, their side effects, including liver dysfunction [13], statin-induced myopathy [14], high creatinine level, high creatine kinase level, cannot be ignored. Hence, effective treatment for dyslipidemia with little side effect has become a major field of interest [15].

Traditional Chinese medicine (TCM) has been used in clinical practice for more than 2000 years in China. Clinical and lab research showed that TCM has certain traits and some strengths in treating dyslipidemia [16–18]. In diagnosing, a TCM doctor would first determine the syndrome (That's the classification or pattern of diseases in TCM), of the patient based on factors for diagnosis such as symptoms, tongue and pulse manifestations, etc., and then treat according to the syndrome (classification). The essence of TCM diagnosis is a classification problem. Previous research [19, 20] has shown that mutual obstruction of phlegm and stasis (MOPS) is the most common dyslipidemia classification. However, Which factors for diagnosis should be used as diagnostic rules and how to quantify their importance all depend on the doctor's personal experience, lacking of unified standards and verification on large sample data. Thus, we hope to develop a new type of prediction tool based on the factors that can diagnose objectively without relying on personal experience.

In recent years, great progress has been achieved in applying deep-learning in medical research. As an approach to deep learning, the artificial neural network (ANN) is a highly parameterized, non-linear model [21]. It can approximate observed outcomes with minimal error [22], or approximate any continuous function [23], and support non-linear complex classification problems. ANN has been widely used in medical research, such as providing decision support in cancer [24], predicting of clinical deterioration in adult patients with hematologic malignancies [25], and tumor biology [26, 27], etc.

**Fig. 1:**
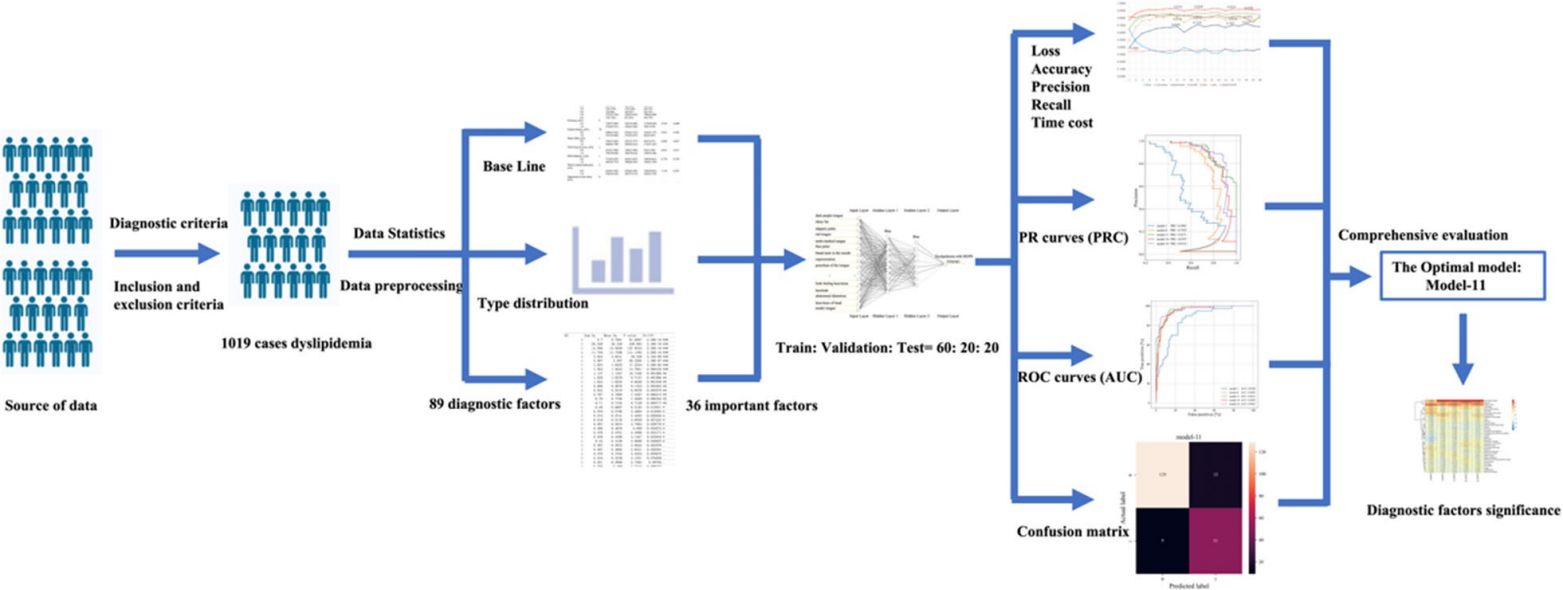
Research flow for data processing and construction of the predictive models. After conducting a preliminary screening of the data collected from the data source, 1019 cases of patients with dyslipidemia were obtained. The baseline table, type distribution, and 89 diagnostic factors were obtained through data statistics and processing. The 89 diagnostic factors were screened using multiple linear regression and were sorted based on their correlation with the output parameters to obtain the 36 most important diagnostic factors. The 36 important diagnostic factors were used as inputs to the models, with whether or not the patient has dyslipidemia with MOPS as the output. The data set was randomly divided into a training set, validation set, and test set in a ratio of 60:20:20, and the models' performance were evaluated. Finally, the optimal model was determined to be Model-11 based on the comprehensive evaluation results of the models, and the importance distribution of the diagnostic factors was calculated

**Fig. 2:**
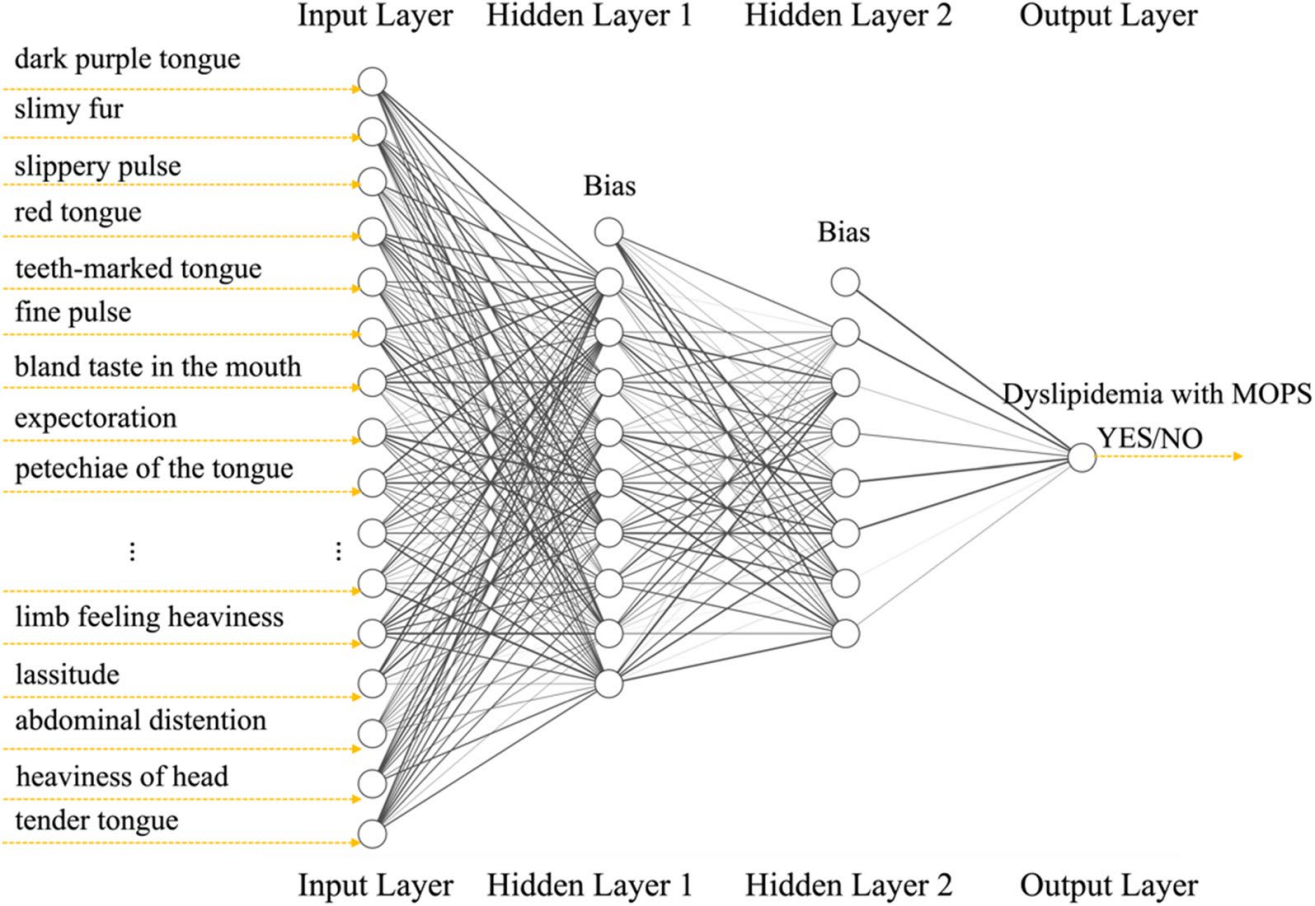
The schematic diagram of the predictive models. The nodes of the neural network are represented by hollow circles, and the weight of the neural network connection is represented by the width of the edges. The input parameters are represented by the 36 nodes of the input layer on the left side of the model, corresponding to the 36 diagnostic factors in TCM. In the middle are hidden layers. On the right is the output layer consisting of one node. The node represents the output parameter, whether the predicted value of the model was dyslipidemia with MOPS

As of now, there is no similar research on the classification and prediction model for dyslipidemia in TCM. In this study, we try to use the deep learning frameworks TensorFlow [28, 29] and Keras [30, 31] to train and construct an effective predictive model for dyslipidemia with MOPS. The TCM diagnosis process was converted into a process of multi-factor classification. A large amount of clinical data was used to develop the models. Research Flow for data processing and construction of the predictive model is illustrated in Fig. 1. Standardized and objective diagnostic patterns buried in data were then revealed, paving the way for more efficient TCM treatment of dyslipidemia (See Fig. 2)

## Methods and materials

### Source of data

This study was based on a cross-sectional data collection. The data came from 1,019 cases of confirmed dyslipidemia collected from 2013 to 2016 by three hospitals, namely Guang'anmen Hospital of Chinese Academy of Traditional Chinese Medicine, Beijing Traditional Chinese Medicine Hospital Affiliated to Capital Medical University and Dongzhimen Hospital of Beijing University of Chinese Medicine. All patients signed informed consent.

### Diagnostic criteria

The diagnostic criteria of dyslipidemia in this study are based on the Guidelines for the Prevention and Treatment of Dyslipidemia in Chinese Adults (2016 Revision) [32], that is, TC ≥ 6.2 mmol /L and / or TG ≥ 2.3 mmol /L and / or LDL-C ≥ 4.1 mmol /L and / or HDL-C < 1.0 mmol /L.

Diagnostic criteria of TCM in this study are based on the differentiation standard of dyslipidemia with MOPS in the Clinical Diagnosis and Treatment Terminology of Traditional Chinese Medicine—Syndrome Part [33]. The study referred to the Terms of Traditional Chinese Medicine [34] for standardization for syndromes that are unclear in the above documents.

### Inclusion and exclusion criteria

Inclusion criteria: (1) The patient meets the diagnostic criteria of the modern clinic and the syndrome differentiation criteria of TCM; (2) The patient is physically and mentally stable; (3) The patient is between 20 and 90 years old; (4) The patient agrees to sign informed consent.

Exclusion criteria: (1) Patients with secondary hyperlipidemia; (2) Patients with chronic consumptive diseases such as malignant tumors and tuberculosis; (3) Patients with serious heart, liver, kidney, hematopoietic system and other primary diseases; (4) Patients with recent surgery or trauma; (5) Patients with mental diseases; (6) Patients who have recently taken hypolipidemic drugs; (7) Patients with other metabolic abnormalities; (8) Patients whose observation data are incomplete and may affect the result evaluation.

### Syndrome type distribution

Two cardiovascular experts with senior professional titles were responsible for determining the syndrome of the patients based on the disease and syndrome differentiation standard of TCM adopted in this study. A total of 1019 cases of dyslipidemia were included, of which 255 cases were identified as the syndrome of MOPS, accounting for 25.02%. The remaining 764 cases were non syndrome of MOPS, representing 74.98 percent of all cases. Among them were 96 cases of Yin blood deficiency syndrome, 90 cases of Qi deficiency and blood stasis syndrome, 76 cases of phlegm stagnation syndrome, 69 cases of spleen deficiency and Qi stagnation syndrome.

### Data preprocessing

We used the EpiData 3.1 software to create a database after preliminarily sorting out the basic information of the patients' clinical medical records, data on diagnostic factors in TCM and data on the syndrome differentiation and classification of diseases. Two doctors input the data independently on two computers to reduce data error. TCM symptom terms of the study were standardized according to the Study on Standardization Status of TCM Terms [35]. Value 1 was assigned to any reported symptom and value 0 is assigned to symptoms that did not appear. Invalid data were cleared out and cases with incomplete records were removed so that a total of 89 diagnostic factors in TCM without missing items were identified, including chest tightness, wheezing, dark purple tongue, etc. Then we used the R package "stats" (version 3.6.3)[36, 37] to run a multiple linear regression analysis on these 89 diagnostic factors to rank them for feature importance and screen out 36 important factors. These factors were then used as input parameters for the following prediction models.

### The architecture of the models

The fully connected ANN for all models was built based on TensorFlow and Keras, which are popular deep learning frameworks. Considering that a large number of parameter inputs may cause the model diagram automatically drawn by the package of R not to be fully displayed, schematic diagrams were used to describe the architecture of the models in this paper.

### Parameters setting of the models

The activation function of the hidden layer is “ReLU” [38] and the activation function of the output layer is “sigmoid” [39]. The weights of the neural network were determined by minimizing the loss value through gradient descent in a process called standard back propagation [40]. The gradient descent algorithm employed was "Adam" [41], and the learning ratio was set to 0.001. Binary cross_entropy (BCE) [42] was set as the loss function (Formula 1), which is often used for binary classification problems [43].

1 $$Loss = - \frac{1}{output\_size}\mathop \sum \nolimits_{i = 1}^{output\_size} y_{i} \cdot {\text{log}}\hat{y}_{i} + \left( {1 - y_{i} } \right) \cdot {\text{log}}(1 - \hat{y}_{i} )$$

The “sklearn” [44], a random number generator in python, was used to randomly divide the data set of 1019 patients. 60% of the data (611 cases) were distributed to the training set, 20% of the data (204 cases) were distributed to the validation set, and 20% of the data (204 cases) were distributed to the test set. The training set optimization was used to tune model parameters. The validation set was used to test the model during training. The test set was used to test the model after training to evaluate the accuracy of the model.

The model.fit function (https://www.tensorflow.org/guide/keras/customizing_what_happens_in_fit) in TensorFlow was used to train models. The parameters were set as follows.

The class weighting scheme [45] is introduced in the training process for handling the imbalanced data. Tensorflow offers a parameter called class_weight in model.fit function that allows to specify the weights for each of the target classes to ensure the predictive performance for imbalanced data. The batch_size in training was set to 128, and the training Epoch was set to 50. To prevent overfitting, the "early stop" method was used, i.e., the training would stop when the accuracy of the verification set did not increase for ten consecutive trainings.

### Data statistics of the models

The performance of the constructed model was evaluated by calculating the performance of the models on the test set. The statistical indicators were the number of true positive samples (TP), false positive samples (FP), true negative samples (TN), false negative samples (FN), loss, accuracy, precision and recall. Based on the above data, the confusion matrixes, PR and ROC curves were drawn, and the value of PRC (area under PR curve) and AUC (area under ROC curve) were calculated.

### Calculating the factor importance of the diagnostic factors

The significance of the 36 factors that had been previously filtered out was calculated and visualized analysis was conducted using the Permutation feature importance (PFI) method[46, 47]. The R packages "pheatmap" [48] and "RColorBrewer" [49] were used to normalize the values and plot heat and cluster diagram for factor significance.

## Results

### Screening of the diagnostic factors in TCM

As mentioned before, we ran a multiple linear regression analysis on these 89 diagnostic factors in TCM with the R package “stats”, screening out 36 significant factors. These 36 factors were used to train and develop the models.

### Baseline situation of base variables and screened diagnostic factors

The mean age of participants at baseline was 64.4 years, and 525 (51.52%) were males. There were 525 males (51.52%) and 494 females (48.48%). The male to female ratio is 1.06:1. 403 (39.55%) had a smoking history, 272 (26.93%) had a drinking history, 640 (63.94%) had a family history, 680(66.80%) had coronary heart disease and 795(78.10%) had hypertension. 445(43.71%) were complicated with diabetes, 392(38.55%) were complicated with cerebral infarction. See Table 1 for details (Table 1: Baseline situation of the 1019 cases with dyslipidemia). (In Table 1, the variable column for smoking has values ranging from 0 to 6. 0 represents smoking within 6 months, 1 represents smoking within 6 months to 5 years, 2 represents smoking within 5–10 years, 3 represents smoking within 10 to 15 years, 4 represents smoking within 15–20 years, 5 represents smoking for over 20 years, and 6 represents an unspecified time period.)

**Table 1:** Baseline situation

Variables	Missing	All(n = 1019)	Non MOPS 0.0 (n = 764)	MOPS 1.0 (n = 255)	Statistic	*p*
Smoking, n(%)	5					
0.0		611(60.256)	483(63.469)	128(50.593)	20.082	0.003
1.0		26(2.564)	20(2.628)	6(2.372)		
2.0		5(0.493)	4(0.526)	1(0.395)		
3.0		19(1.874)	17(2.234)	2(0.791)		
4.0		7(0.690)	5(0.657)	2(0.791)		
5.0		332(32.742)	224(29.435)	108(42.688)		
6.0		14(1.381)	8(1.051)	6(2.372)		
Drinking, n(%)	9					
0.0		738(73.069)	565(74.440)	173(68.924)	2.916	0.088
1.0		272(26.931)	194(25.560)	78(31.076)		
Family history, n(%)	18					
0.0		640(63.936)	475(63.333)	165(65.737)	0.471	0.492
1.0		361(36.064)	275(36.667)	86(34.263)		
With CHD, n(%)	1					
0.0		338(33.202)	255(33.377)	83(32.677)	0.042	0.837
1.0		680(66.798)	509(66.623)	171(67.323)		
With Hypertension, n(%)	1					
0.0		223(21.906)	168(21.990)	55(21.654)	0.013	0.911
1.0		795(78.094)	596(78.010)	199(78.346)		
With Diabetes, n(%)	1					
0.0		573(56.287)	424(55.497)	149(58.661)	0.776	0.378
1.0		445(43.713)	340(44.503)	105(41.339)		
With Cerebral Infarction, n(%)	2					
0.0		625(61.455)	476(62.385)	149(58.661)	1.116	0.291
1.0		392(38.545)	287(37.615)	105(41.339)		
Oppression in the chest, n(%)	0					
0.0		434(42.591)	313(40.969)	121(47.451)	3.286	0.070
1.0		585(57.409)	451(59.031)	134(52.549)		
Asthma, n(%)	0					
0.0		715(70.167)	543(71.073)	172(67.451)	1.198	0.274
1.0		304(29.833)	221(28.927)	83(32.549)		
Shortness of breath, n(%)	0					
0.0		761(74.681)	557(72.906)	204(80.000)	5.089	0.024
1.0		258(25.319)	207(27.094)	51(20.000)		
Forgetfulness, n(%)	0					
0.0		1002(98.332)	749(98.037)	253(99.216)	1.620	0.203
1.0		17(1.668)	15(1.963)	2(0.784)		
Tinnitus, n(%)	0					
0.0		933(91.560)	703(92.016)	230(90.196)	0.819	0.365
1.0		86(8.440)	61(7.984)	25(9.804)		
Limb feeling heaviness, n(%)	0					
0.0		946(92.836)	717(93.848)	229(89.804)	4.702	0.030
1.0		73(7.164)	47(6.152)	26(10.196)		
Heaviness of head, n(%)	0					
0.0		996(97.743)	751(98.298)	245(96.078)	4.271	0.039
1.0		23(2.257)	13(1.702)	10(3.922)		
Abdominal distention, n(%)	0					
0.0		933(91.560)	692(90.576)	241(94.510)	3.829	0.050
1.0		86(8.440)	72(9.424)	14(5.490)		
Vomit, n(%)	0					
0.0		910(89.303)	690(90.314)	220(86.275)	3.266	0.071
1.0		109(10.697)	74(9.686)	35(13.725)		
Acid reflux and heartburn, n(%)	0					
0.0		871(85.476)	663(86.780)	208(81.569)	4.183	0.041
1.0		148(14.524)	101(13.220)	47(18.431)		
Bland taste in the mouth, n(%)	0					
0.0		1016(99.706)	764(100.000)	252(98.824)	nan	nan
1.0		3(0.294)	0(0.000)	3(1.176)		
Hot flashes and night-time sweating, n(%)	0					
0.0		980(96.173)	730(95.550)	250(98.039)	3.219	0.073
1.0		39(3.827)	34(4.450)	5(1.961)		
Abdominal fullness, n(%)	0					
0.0		979(96.075)	729(95.419)	250(98.039)	3.481	0.062
1.0		40(3.925)	35(4.581)	5(1.961)		
Cough, n(%)	0					
0.0		700(68.695)	555(72.644)	145(56.863)	22.141	< 0.001
1.0		319(31.305)	209(27.356)	110(43.137)		
Expectoration, n(%)	0					
0.0		747(73.307)	593(77.618)	154(60.392)	28.991	< 0.001
1.0		272(26.693)	171(22.382)	101(39.608)		
Sallow complexion, n(%)	0					
0.0		1002(98.332)	754(98.691)	248(97.255)	2.404	0.121
1.0		17(1.668)	10(1.309)	7(2.745)		
Lassitude, n(%)	0					
0.0		626(61.433)	455(59.555)	171(67.059)	4.544	0.033
1.0		393(38.567)	309(40.445)	84(32.941)		
Aversion to cold, n(%)	0					
0.0		925(90.775)	684(89.529)	241(94.510)	5.665	0.017
1.0		94(9.225)	80(10.471)	14(5.490)		
Puffy face and swollen limbs, n(%)	0					
0.0		946(92.836)	714(93.455)	232(90.980)	1.761	0.184
1.0		73(7.164)	50(6.545)	23(9.020)		
Thin tongue, n(%)	0					
0.0		1011(99.215)	756(98.953)	255(100.000)	nan	nan
1.0		8(0.785)	8(1.047)	0(0.000)		
Teeth-marked tongue, n(%)	0					
0.0		911(89.401)	669(87.565)	242(94.902)	10.860	< 0.001
1.0		108(10.599)	95(12.435)	13(5.098)		
Fissured tongue, n(%)	0					
0.0		943(92.542)	702(91.885)	241(94.510)	1.909	0.167
1.0		76(7.458)	62(8.115)	14(5.490)		
Tender tongue, n(%)	0					
0.0		1009(99.019)	754(98.691)	255(100.000)	nan	nan
1.0		10(0.981)	10(1.309)	0(0.000)		
Red tongue, n(%)	0					
0.0		649(63.690)	440(57.592)	209(81.961)	49.096	< 0.001
1.0		370(36.310)	324(42.408)	46(18.039)		
Dark purple tongue, n(%)	0					
0.0		429(42.100)	421(55.105)	8(3.137)	211.817	< 0.001
1.0		590(57.900)	343(44.895)	247(96.863)		
Slimy fur, n(%)	0					
0.0		566(55.545)	503(65.838)	63(24.706)	130.993	< 0.001
1.0		453(44.455)	261(34.162)	192(75.294)		
Petechiae of the tongue, n(%)	0					
0.0		964(94.603)	730(95.550)	234(91.765)	5.364	0.021
1.0		55(5.397)	34(4.450)	21(8.235)		
White fur, n(%)	0					
0.0		522(51.227)	407(53.272)	115(45.098)	5.113	0.024
1.0		497(48.773)	357(46.728)	140(54.902)		
Slippery pulse, n(%)	0					
0.0		644(63.199)	561(73.429)	83(32.549)	137.378	< 0.001
1.0		375(36.801)	203(26.571)	172(67.451)		
Uneven pulse, n(%)	0					
0.0		997(97.841)	745(97.513)	252(98.824)	1.554	0.213
1.0		22(2.159)	19(2.487)	3(1.176)		
Fat tongue, n(%)	0					
0.0		913(89.598)	680(89.005)	233(91.373)	1.150	0.284
1.0		106(10.402)	84(10.995)	22(8.627)		
Fine pulse, n(%)	0					
0.0		685(67.223)	478(62.565)	207(81.176)	30.055	< 0.001
1.0		334(32.777)	286(37.435)	48(18.824)		
Fixed pain, n(%)	0					
0.0		968(94.995)	722(94.503)	246(96.471)	1.557	0.212
1.0		51(5.005)	42(5.497)	9(3.529)		
Distension in the hypochondriac region, n(%)	0					
0.0		996(97.743)	744(97.382)	252(98.824)	1.800	0.180
1.0		23(2.257)	20(2.618)	3(1.176)		
Depression, n(%)	0					
0.0		1015(99.607)	760(99.476)	255(100.000)	nan	nan
1.0		4(0.393)	4(0.524)	0(0.000)		
Vexation, n(%)	0					
0.0		996(97.743)	744(97.382)	252(98.824)	1.800	0.180
1.0		23(2.257)	20(2.618)	3(1.176)		
Gender, n(%)	0					
Male		525(51.521)	365(47.775)	160(62.745)	17.155	< 0.001
Female		494(48.479)	399(52.225)	95(37.255)		
Heart rate, mean(± SD)	18	76.371(± 12.626)	76.336(± 12.818)	76.472(± 12.036)	-0.148	0.883
Body weight, mean(± SD)	110	70.432(± 12.677)	69.753(± 12.564)	72.461(± 12.798)	-2.800	0.005
Height, mean(± SD)	73	164.611(± 8.949)	164.325(± 8.411)	165.470(± 10.354)	-1.704	0.089
Age, mean(± SD)	2	64.445(± 12.540)	63.849(± 12.677)	66.236(± 11.945)	-2.634	0.009

*T test was used for heart rate, weight, height and age. Chi-square test was used for other items. Data are presented as number (%). Abbreviations: CHD Coronary heart disease

### Model construction for the prediction of dyslipidemia with MOPS

**Fig. 3:**
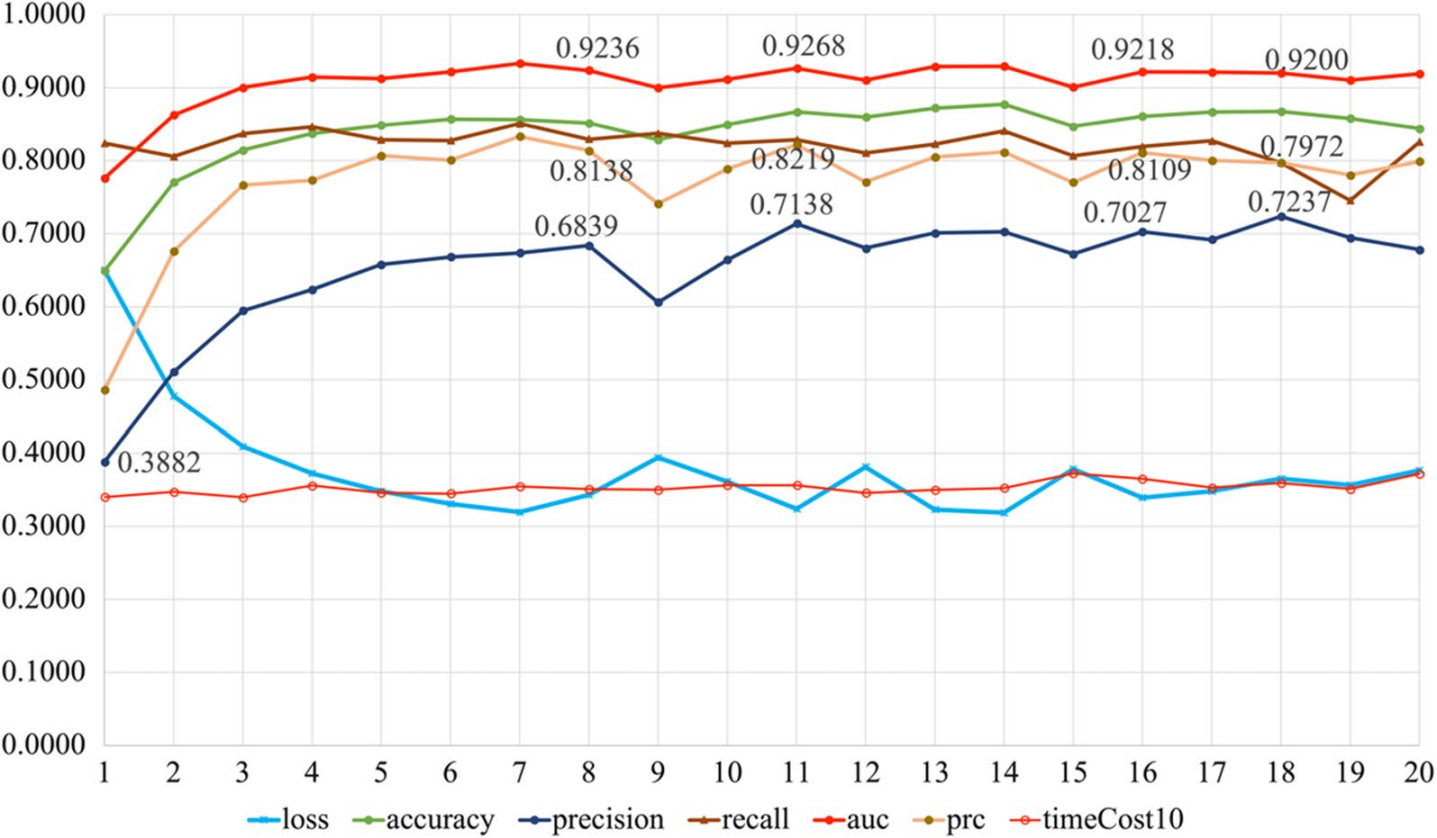
The training results of the 20 predictive models. The curves in the figure represent the loss, accuracy, precision, recall, AUC, PRC, and timeCost10 for model-1 to model-20, respectively

The number of neurons of each model can be seen on Table 2. We calculated performance indicators of the models such as accuracy, precision, recall, AUC, PRC and time cost for ten training sessions (timeCost10). Each model was trained ten times with data set randomly divided. The above indicators of each model were averaged to evaluate the impact of the number of hidden layers and neurons on the models’ performance, as shown in Fig. 3.

**Table 2:** Structure of the predictive models. The hidden layers of the models were set to 0, 1, 2, 3, and 4, respectively, and the number of neurons in each hidden layer was set to 0, 8, 16, 32, 64, 128, et al.

Model	Hidden-layer number	Neuron number in each hidden layer	Model	Hidden-layer number	Neuron number in each hidden layer
1	0	0	11	2	64–32
2	1	8	12	2	128–32
3	1	16	13	2	256–32
4	1	32	14	2	512–32
5	1	64	15	3	32–16-8
6	1	128	16	3	64–32-16
7	1	256	17	3	128–32-16
8	1	512	18	4	64–32-16–8
9	2	16–8	19	4	128–32-16–8
10	2	32–16	20	4	256–32-16–8

As is shown in Fig. 3, from model-1 to model-5, accuracy, precision, recall, auc, and prc kept increasing while loss kept decreasing. And from model-5 to model-20, these indicators stayed steady. Time cost for ten trainings mostly remained steady across models. From model-1 to model-5, the number of hidden layers of the model increases from 0 to 1, and the number of neurons in the hidden layer also increases, which indicates that the model with 0 hidden layers does not predict as well as the model with 1 hidden layer, and within a certain range of the number of neurons in the hidden layer, the increase in the number of neurons helps to improve the prediction of the model. However, as the number of hidden layers and hidden layer neurons continues to increase, the performance of the model does not continue to improve but remains at a high level and does not increase. In general, for model training using TensorFlow, the training sample size needs to be adapted to the number of hidden layers and neurons, and it is not advisable to use complex neural network structures for training small samples, and similarly, it is not advisable to use simple neural network structures for training large samples. Figure 3 shows that models 5–20 are more appropriate with the sample size of 1019 cases used in this study. So we will evaluate and select the optimal model from model-5 to model-20.

### Performance analysis of the prediction models for dyslipidemia with MOPS

The models were divided into five groups based on their number of hidden layers (0, 1, 2, 3, 4). Then we selected the model with the highest precision in each group: model-1, model-8, model-11, model-16 and model-18. Using the training data with optimal loss value, we evaluated the performance of the five models in the test set in 50 Epochs of training.

**Fig. 4:**
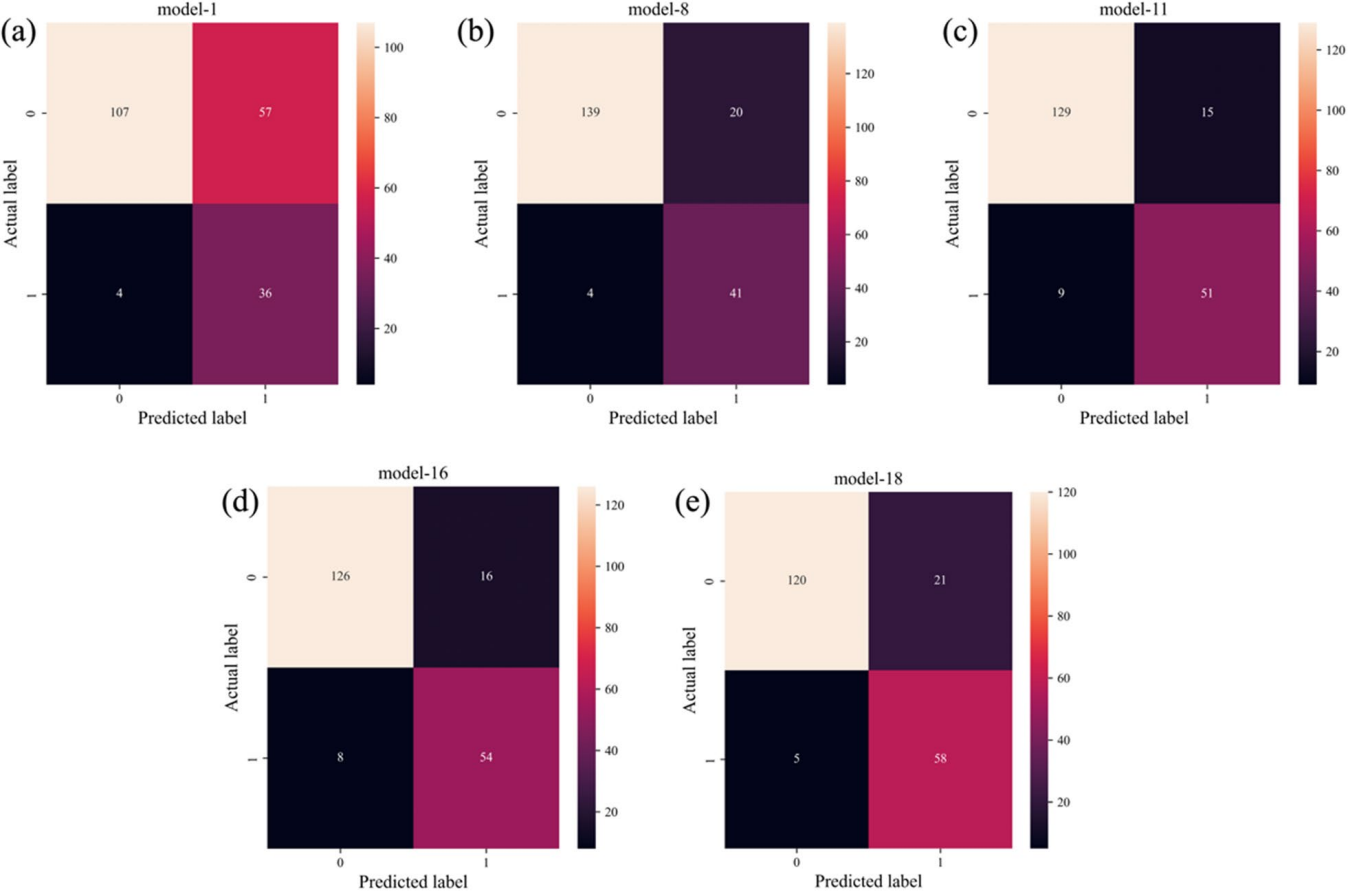
Confusion matrix of the five models **a** Confusion matrix of model-1 **b** Confusion matrix of model-8 **c** Confusion matrix of model-11 **d** Confusion matrix of model-16 **e** Confusion matrix of model-18 (Note: The upper left corner of the confusion matrix represents the number of TN predicted by the model, the lower right corner represents the number of TP, the upper right corner represents the number of FP, and lower left corner represents the number of FN)

We calculated the confusion matrix of each model, as shown in Fig. 4. The sum of TN and TP samples meant the number of correct predictions by the model. Model-1, with 0 hidden layers, had 143 TN and TP samples (Fig. 4a). Model-8, with 1 hidden layer, had 180 TN and TP samples (Fig. 4b). Model-11, with 2 hidden layers, had 180 TN and TP samples (Fig. 4c). Model-16, with 3 hidden layers, also had 180 TN and TP samples (Fig. 4d). Model-18, with 4 hidden layers had 178 TN and TP samples (Fig. 4e). These results indicate that models with at least one hidden layer have better prediction performance than those with 0 hidden layer.

**Fig. 5:**
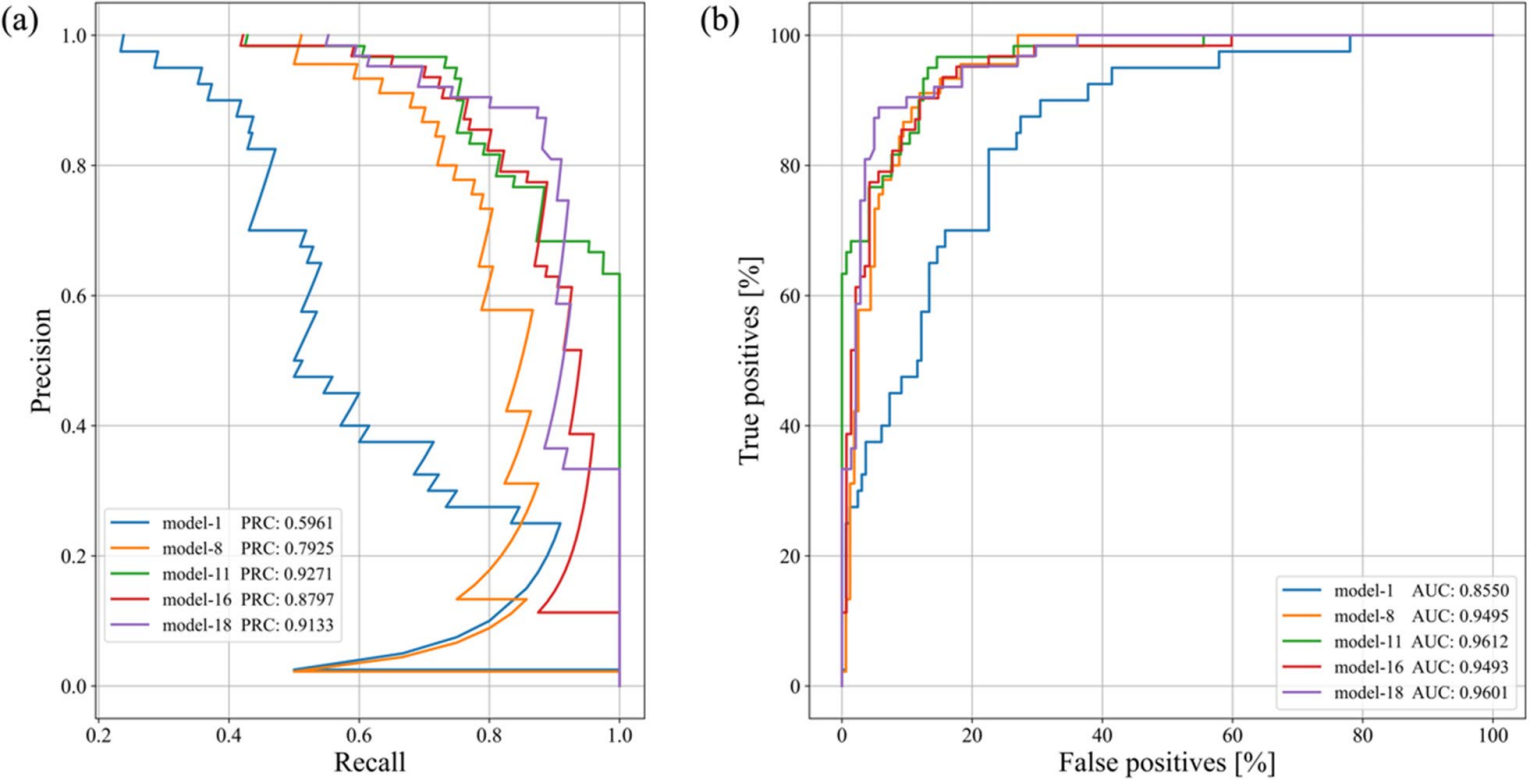
**a** PR curves (PRC) of the 5 models **b** ROC curves (AUC) of the 5 models

We plotted PR curves and ROC curves to evaluate the prediction performance of the models. As shown in Fig. 5a, b, the closer the PR curve is to the right, the better the model's performance; the closer the ROC curve is to the left, the better the model's performance [50]. Furthermore, the discrimination ability of the models was evaluated by the area under the curve of the PR and ROC. PRC is the area under the PR curve. AUC is the area under the ROC curve. When the values of PRC and AUC are greater than 0.5, it means the model performs well. The closer these values are to 1, the better the model's performance [50, 51]. The PRC and AUC values show that all the five models performed well, while Model-11 performed the best. In summary, all the five models had good prediction performance. Among them, those with multiple hidden layers performed better than those with 0 hidden layer. And Model-11 was optimal in performance.

### The significance analysis of diagnostic factors for dyslipidemia with MOPS

**Fig. 6:**
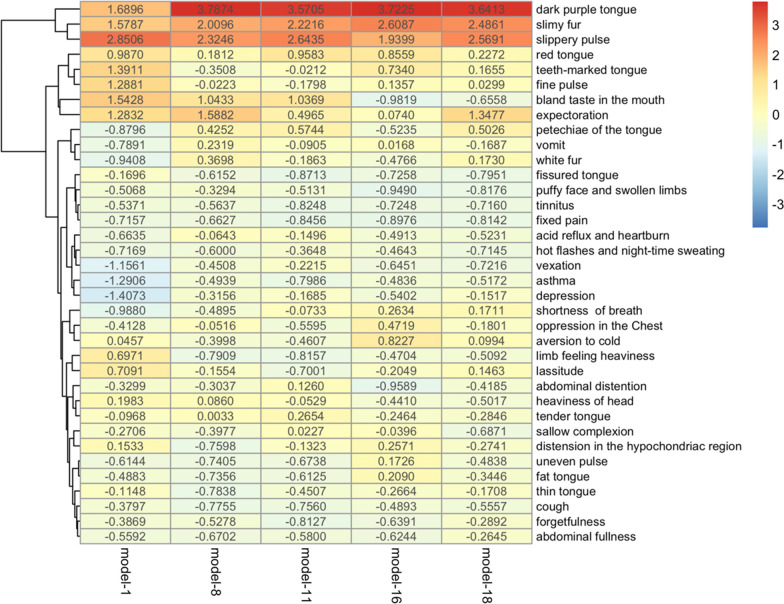
Heat and cluster diagram of diagnostic factors on five screened models *Note* the number in the box is the value obtained by normalizing the importance value of the diagnostic factor calculated using PFI, the value ∈ [− 4, 4)

In this study, the above five models were used to evaluate the significance of diagnostic factors for dyslipidemia with MOPS. We calculated the significance values of the diagnostic factors with PFI. The results are shown in Fig. 6. From the heat diagram, it can be seen that diagnostic factors, such as dark purple tongue, slippery pulse, slimy fur, expectoration, petechiae of the tongue, were of high significance. From the hierarchical cluster diagram, it can be seen that dark purple tongue, slippery pulse, slimy fur, expectoration and so on were on the higher hierarchy, indicating greater importance. According to this, we believe that standardized and objective diagnostic rules for dyslipidemia with MOPS can be constructed based on dark purple tongue, slippery pulse, slimy fur, expectoration, petechiae of the tongue.

It is worth mentioning that in the clinical diagnosis scale of MOPS compiled by Fang Ge et al. [52], dark purple tongue, slippery pulse and slimy fur are also in the high-frequency vocabulary of this syndrome. It means that from the perspective of clinical observation, dark purple tongue, slippery pulse and slimy fur are also of great significance in the differentiation and classification of dyslipidemia with MOPS, which is highly consistent with the prediction results of the models.

## Discussion

Dyslipidemia has drawn extensive attention due to its important clinical significance. There is no disease term for dyslipidemia in TCM, but it is currently considered to be close to the concepts of “phlegm turbidity” and “cream fat” in TCM. Although dyslipidemia has a variety of classification in TCM, its basic pathological characteristics are phlegm and blood stasis as manifestation, deficiency as the root cause, and mutual obstruction of phlegm and blood stasis as the problem [19]. This description is also consistent with this study's finding that MOPS is the most frequent among all the classifications.

The process of diagnosis in TCM is essentially a classification problem. However, TCM diagnosis relies heavily on the doctors' experience, which is highly subjective. Standardized diagnostic rules cannot be formed, which makes it difficult to standardize and promote the characteristics of TCM diagnosis and treatment.

This study used deep learning to train and construct a prediction model of dyslipidemia based on the clinical data in TCM, so as to confirm the feasibility of inputting the diagnostic factors in TCM (such as dark purple tongue, chest tightness, etc.) into the model to predict whether the patient has dyslipidemia with MOPS, simulating the process of clinical syndrome classification of dyslipidemia in TCM.

One advantage of this model is that it can help solve the problem of the lack of objectivity of TCM in clinical diagnosis. Another significant advantage of this model is the high accuracy, which will reduce the workload caused by clinical misdiagnosis. In addition, the model efficiently uncovers and utilizes the hidden rules and patterns buried in a large amount of clinical data so that standardized and objective diagnostic rules for dyslipidemia with MOPS can be constructed.

To sum up, in this study, we constructed prediction models for dyslipidemia with MOPS through deep learning method with a large amount of multi-centered clinical data. We further evaluated the performance of the models. Results of the study show that the models performed well in predicting dyslipidemia with MOPS, and the model-11 is the optimal model. In the meantime, diagnostic factors in TCM, such as dark purple tongue, slippery pulse and slimy fur, were screened out as significant factors and diagnostic rules for the diagnosis of MOPS. The study is an avant-garde attempt at introducing the deep-learning method into the research of TCM, contributing to the standardization and objectiveness of TCM diagnosis for dyslipidemia.

### Strengths and limitations

As far as we know, this study is the first to use a diagnostic model based on deep learning to predict whether patients have dyslipidemia with MOPS, so as to guide the corresponding treatment in TCM. Although this is just a small step ahead, it reveals the great potential in applying the deep learning method to clinical data mining and diagnosis, which will help clinicians reduce subjectivity and improve stability in clinical diagnosis.

Unlike traditional linear models, the prediction model based on deep learning is a nonlinear “black box”. Although the “black box” can provide more accurate prediction results, its opaqueness and lack of clinical interpretability may lead to some restrictions to applying the deep learning method. In addition, due to personnel and funds limitations, we cannot collect more data for external verification. In the next research, we plan to carry out clinical data collection of larger samples to verify further, improve and modify our prediction model for even stronger prediction performance.

## Conclusions

This study proved the feasibility of constructing a diagnostic prediction model based on the deep learning method to predict whether patients have dyslipidemia with MOPS to guide the corresponding TCM treatment. The model-11 is the optimal model, with a high level of accuracy, and it provides clinicians with a more objective and stable guide for diagnosing and treating dyslipidemia with MOPS in TCM.

## Data Availability

The data and materials of this study will be made available by the corresponding author upon a reasonable request.
